# Biopsychosocial Influences on the Gut Microbiome in Women’s Health: Moving Towards Eubiosis

**DOI:** 10.3390/bs16050627

**Published:** 2026-04-22

**Authors:** Ashley J. Blount, Kara Schneider, Abby L. Bjornsen, Thang S. Tran, Gurudutt Pendyala, Tiffany A. Moore

**Affiliations:** 1 Department of Counseling, University of Nebraska Omaha, Omaha, NE 68182, USA; karaschneider@unomaha.edu (K.S.); abjornsen@unomaha.edu (A.L.B.); tstran@unomaha.edu (T.S.T.); 2 Department of Anesthesiology, University of Nebraska Medical Center, Omaha, NE 68198, USA; gpendyala@unmc.edu; 3 College of Nursing, University of Nebraska Medical Center, Omaha, NE 68198, USA; tamoore@unmc.edu

**Keywords:** women’s health, mental health, microbiome, biopsychosocial, treatment, psythotherapy, eubiosis

## Abstract

Research on the human microbiome, particularly the gut microbiome, has expanded rapidly as its influence on health and behavior becomes increasingly evident. Once understood primarily in terms of digestion and immune function, the microbiome is now recognized as a key contributor to brain function, mood regulation, and social behavior. Emerging evidence links microbial dysbiosis to the onset and persistence of mood disorders, opening new pathways for mental health research and intervention. This paper challenges reductionist biomedical models by advancing a biopsychosocial framework for interpreting health outcomes related to microbiome dynamics. The gut–brain axis illustrates the biological complexity of these interactions, with microbial communities shaping neurodevelopment and neurotransmitter production. Psychologically, alterations in microbial composition have been associated with depression, anxiety, and stress responsivity, while social determinants—including early-life environments, socioeconomic conditions, and relationships—structure microbial variation in ways that may reinforce existing health inequities. Focusing on women’s health, this narrative review examines how microbial states both influence and are shaped by interconnected biological, psychological, and social factors. Interdisciplinary implications of microbiome research for understanding and achieving eubiosis and holistic models care in both research and clinical practice are discussed.

## 1. Introduction

The microbiome has emerged as a critical focus of interdisciplinary research, highlighting the profound influence of microbial communities on human health and behavior. Comprising trillions of microorganisms residing primarily in the gut, the microbiome is now recognized not only for its role in digestion and immunity but also for its effects on brain function, mood, and social behavior (Cryan et al., 2019; Morais et al., 2021). Further, the collective evidence points to gut dysbiosis, or the imbalance in gut microbiome communities, not merely as an associated feature but as a potential driving factor in the emergence and persistence of mood disorders, highlighting the gut microbiome as a promising focus for mental health research and treatment (Mehta, 2025; Simpson et al., 2021; Valles-Colomer et al., 2019). This growing body of evidence challenges traditional biomedical models by underscoring the necessity of a biopsychosocial approach focusing on eubiosis or the state of balanced and healthy microbiome (Mayer et al., 2022).

Biologically, the gut–brain axis illustrates a complex communication network between the central nervous system and the enteric microbiota, influencing neurodevelopment and neurotransmitter production (Cryan et al., 2019; Mayer et al., 2014; Morais et al., 2021). Recent scientific developments have underscored the importance of the brain–gut-immune axis in both health and disease, highlighting bidirectional signaling pathways involving neural, endocrine, and inflammatory mechanisms (Agirman & Hsiao, 2021). The gastrointestinal (GI) tract is home to the enteric nervous system, often referred to as the “second brain,” due to its extensive network of neurons and complex interactions with the central nervous system (Mayer et al., 2022; Rhee et al., 2009). Within this system, the microbiome plays a pivotal role in the bidirectional communication between the gut and the brain (Dinan & Cryan, 2017). Dysbiosis has been associated with both acute and chronic disease conditions, highlighting its role as a central factor in human health (Cryan et al., 2019). Understanding the complexity of biopsychosocial factors in health and disease, the brain–gut axis is central to this understanding.

Psychologically, variations in microbial composition have been associated with mental and emotional concerns such as depression, anxiety, and other stress-related disorders (Dinan & Cryan, 2017; Simpson et al., 2021). Alterations in microbial diversity and metabolite production can influence neurotransmitter synthesis, including serotonin, gamma-aminobutyric acid (GABA), and dopamine, which play central roles in mood regulation and emotional processing (Morais et al., 2021). Chronic psychological stress has been shown to disrupt gut permeability and increase systemic inflammation, potentially contributing to dysbiosis and reinforcing symptom severity (Agirman & Hsiao, 2021). Conversely, imbalances in the gut microbiota may amplify stress responsivity through dysregulation of the hypothalamic–pituitary–adrenal (HPA) axis, further linking microbial health to psychological resilience and vulnerability (Cryan et al., 2019). Emerging evidence suggests that psychotherapeutic interventions, stress reduction practices, and behavioral modifications may indirectly influence microbial composition by altering neuroendocrine and inflammatory pathways (Mayer et al., 2022). Together, these findings position the microbiome as both a mediator and a reflection of psychological health, highlighting its relevance within an integrated biopsychosocial framework.

Socially, factors like sociocultural influences, socioeconomic status, and interpersonal relationships shape the microbiome in ways that may perpetuate health disparities (Heiman & Greenway, 2016; Johnson & Foster, 2018; Schnorr et al., 2014). The microbiome is deeply embedded within the environmental and relational contexts that shape human life, reflecting patterns of interaction, inequality, and culture across the lifespan (Amato et al., 2021). Dietary practices, food accessibility, sanitation, urbanization, antibiotic exposure, and early-life caregiving environments all influence microbial colonization and diversity, often in ways that mirror broader social structures and disparities (Dominguez-Bello et al., 2019; Heiman & Greenway, 2016; Johnson & Foster, 2018). Socioeconomic status has been linked to differences in nutritional quality, chronic stress exposure, and healthcare access—factors that can alter microbial composition and contribute to uneven health outcomes across populations (Miller et al., 2017). Cultural traditions surrounding food preparation, communal living, and childbirth practices further modulate microbial transmission and resilience (Dominguez-Bello et al., 2019; Schnorr et al., 2014). Moreover, social stressors such as discrimination, social isolation, and community instability may indirectly influence the microbiome through neuroendocrine and inflammatory pathways, reinforcing the interconnectedness of social experience and biological function (Miller et al., 2017). Recognizing these influences underscores the necessity of integrating social determinants of health into microbiome research, as microbial balance cannot be fully understood or restored without attention to the societal contexts in which individuals live.

Coupled with female-specific hormonal and reproductive life stages (e.g., menstrual cycle, pregnancy, menopause) and psychotherapeutic modulators (e.g., counseling, therapy), the biopsychosocial factors influencing gut microbiota are uniquely dynamic and context-dependent, reflecting the intricate interplay between endocrine fluctuations, psychological processing, and social experience across the female lifespan (Jašarević et al., 2016; Nuriel-Ohayon et al., 2016). As such, this paper explores the theoretical framework of the biopsychosocial model and its influences of the human microbiome related to women’s health (Table 1), examining how microbial health is both shaped by and influenced biological processes, psychological states, and social environments. Through this lens, we highlight the interdisciplinary implications of microbiome research to find eubiosis, or a healthy gut microbiome, and advocate for a holistic women’s health model in both research and clinical practice. Henceforth, we use the terms “female,” “women” and “women’s health” interchangeably.

## 2. Biopsychosocial Factors Influencing Gut Microbiota

### 2.1. Biological Factors

#### 2.1.1. Physical and Nutritional

Traditional biological factors influencing gut microbiota include nutritional and physical components. Nutrition and metabolism are key determinants of gut microbial composition and overall gut function. Dietary patterns significantly influence microbial diversity, with diets rich in fiber, plant-based foods, and polyphenols promoting the growth of beneficial bacteria, whereas high-fat or high-sugar diets are associated with reduced microbial diversity and increased prevalence of pro-inflammatory species (Singh et al., 2017; Valdes et al., 2018; Zmora et al., 2018). Microbial metabolites, such as short-chain fatty acids produced through the fermentation of dietary fibers, play a critical role in maintaining intestinal barrier integrity, modulating immune responses, and supporting metabolic and neuroendocrine health (Koh et al., 2016). Consequently, nutrition and metabolic status not only shape gut microbiota composition but also impact systemic health outcomes, highlighting the importance of diet as a modifiable factor in promoting gut homeostasis and overall well-being (Valdes et al., 2018; Zmora et al., 2018).

Regarding physical health, factors influencing gut microbiota encompass the inherent physiological and anatomical characteristics of the host that shape microbial colonization and stability. Genetics play a foundational role by influencing immune responses, mucosal barrier integrity, and the production of antimicrobial peptides, all of which regulate microbial composition (Goodrich et al., 2014). Age is another critical determinant, as the microbiome undergoes dynamic shifts from infancy through adulthood and into older age, reflecting developmental, hormonal, and immunological changes (Odamaki et al., 2016). Gastrointestinal structure and function—including gastric acid secretion, intestinal motility, mucus production, and epithelial permeability—create selective environments that either support or inhibit specific microbial populations (Donaldson et al., 2016). Immune system activity further modulates microbial balance by distinguishing between commensal and pathogenic organisms, maintaining tolerance while preventing overgrowth (Belkaid & Hand, 2014). Additionally, factors such as circadian rhythms, sleep patterns, and systemic inflammation can alter gut physiology in ways that indirectly affect microbial diversity and resilience (Thaiss et al., 2016). Collectively, these physical biological determinants provide the structural and regulatory framework within which the gut microbiota exists and adapts.

#### 2.1.2. Reproductive Life Stages

The unique composition of women’s bodies makes biological factors a primary concern for gut microbiota. Within this biological realm, several hormonal influences on the gut microbiota exist that are unique based on biological females. The menstrual cycle, for example, offers fluctuations in the gut microbial composition (Santos-Marcos et al., 2018, 2019). There are also pregnancy-associated microbiome changes (Koren et al., 2012) as well as menopause effects (Chen & Madak-Erdogan, 2016). Hormonal fluctuations across reproductive life stages have effects on gut microbial composition, with implications for metabolic, immune, and neuroendocrine function. During the menstrual cycle, changes in estrogen and progesterone levels influence gut physiology, including motility, mucosal immunity, and nutrient absorption. Santos-Marcos et al. (2018) reported that microbial composition varies across menstrual phases, with fluctuations in the abundance of beneficial bacteria such as Bifidobacterium and Lactobacillus. Further, these cyclical microbial changes may contribute to variations in gastrointestinal symptoms, mood, and metabolic function throughout the cycle.

Pregnancy represents another period of substantial microbial and hormonal adaptation. Koren et al. (2012) found that, during the third trimester, women exhibit decreased microbial diversity and increased relative abundance of Proteobacteria and Actinobacteria, which are associated with enhanced energy extraction and low-grade inflammation. These microbial shifts are thought to support female metabolic adaptations necessary for fetal growth but may also contribute to gestational complications, including excessive weight gain and gestational diabetes (Chen & Madak-Erdogan, 2016).

Menopause is characterized by the decline of estrogen production, which also influences gut microbial communities. Chen and Madak-Erdogan (Chen & Madak-Erdogan, 2016) observed that postmenopausal women often experience reduced abundance of beneficial microbes and increased prevalence of pro-inflammatory bacteria. Such changes may contribute to systemic inflammation, metabolic dysregulation, and heightened risk for age-related chronic conditions (Chen & Madak-Erdogan, 2016). The decline in estrogen’s modulatory effects on the gut may additionally impact gut barrier integrity and microbial metabolite production, further influencing overall health outcomes (Chen & Madak-Erdogan, 2016).

Collectively, these findings illustrate that reproductive life stages—from menstruation to pregnancy to menopause—are associated with dynamic shifts in the gut microbiome. These shifts not only reflect physiological adaptations to hormonal changes but also influence broader aspects of health, underscoring the importance of considering sex-specific and life stage–specific factors in gut microbiome research and women’s health interventions.

#### 2.1.3. Immune Function

Immune function and inflammation are critical factors influencing the gut microbiota within the biological realm. The brain–gut-immune axis provides important insights into how stress and behavior are linked with biological processes. Stress, through activation of the hypothalamic–pituitary–adrenal (HPA) axis, alters the composition of gut microbiota and compromises intestinal integrity (Cryan et al., 2019; Mayer et al., 2022). This disruption leads to increased intestinal permeability, or “leaky gut,” which permits microbial components to cross into systemic circulation and stimulate immune responses (Dinan & Cryan, 2017). In turn, this cascade of events can influence neuroinflammatory processes, stress reactivity, and overall mental health (Agirman & Hsiao, 2021).

Microbiota communicate with the central nervous system through multiple interconnected pathways, including immune activation, the vagus nerve, tryptophan metabolism, microbial metabolites, neurometabolites, and bacterial cell wall components (Cryan et al., 2019; Dinan & Cryan, 2017). These pathways demonstrate how alterations in microbial composition can shape stress responses, mood regulation, and behavior. While past interventions have primarily focused on dietary modification, pharmacological approaches, or probiotic supplementation, emerging perspectives suggest that addressing chronic stress through counseling and psychotherapy may also represent a novel method of modulating the gut microbiome (Mayer et al., 2022; Schnorr et al., 2014). This approach aligns with the growing recognition of the integrative nature of the brain–gut-immune axis and its potential as a target for improving both physical and psychological health.

Sex-specific immune responses further influence gut health. Females typically exhibit stronger innate and adaptive immune activity compared to males, which can affect microbial composition, intestinal barrier function, and susceptibility to inflammation-related conditions (Klein & Flanagan, 2016). These immunological differences may help explain sex-based variations in the prevalence of autoimmune disorders, gastrointestinal diseases, and microbiota-associated metabolic or mood disturbances. Recognizing the interplay between the gut microbiota, immune regulation, and sex-specific immune responses is essential for understanding individual differences in gut health and for developing targeted interventions that account for biological sex.

### 2.2. Psychological Factors

The gut–brain axis functions as a bidirectional communication network that plays a central role in mood regulation and emotional health. Foster et al. (Foster et al., 2021) emphasized that this system operates through multiple interconnected pathways, including the neural route via the vagus nerve, the endocrine route through the HPA axis, the immune system through cytokine signaling, and the metabolic route involving microbial metabolites such as short-chain fatty acids (Cryan et al., 2019; Foster et al., 2021; Morais et al., 2021). The vagus nerve in particular serves primarily as an afferent pathway, with approximately 80% of its “information carrying” occurs from the gut to the brain (Bonaz et al., 2018; Breit et al., 2018). This communication is crucial for homeostasis and influences a myriad of bodily functions, including appetite regulation, mood, and emotional responses (Breit et al., 2018; Cork, 2018; Cryan et al., 2019; Mayer et al., 2022). These mechanisms enable the gut microbiota to influence central nervous system activity while also allowing psychological states and stress to alter gut microbial composition, creating a dynamic feedback loop (Morais et al., 2021).

Disruptions within this communication system have been shown to significantly impact emotional well-being. For instance, alterations in microbial communities can influence neurotransmitter synthesis, including serotonin and gamma-aminobutyric acid (GABA), which are essential for mood regulation (Foster et al., 2021; Morais et al., 2021). Vagus nerve stimulation (VNS) in animal model and clinical studies has demonstrated improvements in depressive and anxiety-related symptoms, potentially through modulation of serotonergic and limbic signaling pathways (Bonaz et al., 2018; Breit et al., 2018; Lauer, 2025; Mori et al., 2018). Additionally, the gut microbiota can lead to the modulation of vagal communication from the gut to the brain, influencing gastrointestinal functions and immune responses. For example, elevated levels of lipopolysaccharides can provoke inflammatory signaling that contributes to depressive-like phenotypes via vagal and immune-mediated pathways (Maes et al., 2019; Zhang et al., 2020). Researchers have also found that therapeutic interventions aimed and enhancing vagal tone may also be appropriate for addressing mental health disorders (Bairamian et al., 2022; Bonaz et al., 2018). Conversely, chronic stress can compromise gut barrier integrity, increase intestinal permeability, and shift microbial composition, thereby reinforcing cycles of dysbiosis and emotional dysregulation (Cryan et al., 2019; Mayer et al., 2022). This reciprocal relationship suggests that both gut and brain function must be considered in the study of mood disorders.

Evidence from animal and human studies further supports this bidirectional model. Rodent studies demonstrate that fecal microbiota transplantation from individuals with anxiety or depression can induce corresponding behavioral changes in the animals, providing causal evidence for microbial influence on mood (Lauer, 2025; Morais et al., 2021). Similarly, human studies reveal that individuals with depression or heightened anxiety often exhibit altered microbial diversity compared to healthy controls (Simpson et al., 2021; Valles-Colomer et al., 2019). These findings suggest that therapeutic approaches targeting the microbiome—through stress reduction—may positively influence both gut and brain health.

#### 2.2.1. Mental and Emotional Health

One area of growing interest is the interaction between the HPA axis and the gut–brain–microbiota axis. Dysregulation of the HPA axis, often triggered by psychological stress, can influence gut microbiota composition and, in turn, affect mental health (Cryan et al., 2019; Louwies et al., 2019; Mayer et al., 2014; Morais et al., 2021). Prolonged elevations in cortisol can impair gut barrier function, increasing intestinal permeability and allowing bacterial endotoxins, such as lipopolysaccharides, to enter systemic circulation (Mayer et al., 2014, 2022). This process drives low-grade inflammation and contributes to microbial dysbiosis, which in turn alters the production of metabolites important for brain function and mood regulation (Maes et al., 2019; Moloney et al., 2016; Nuriel-Ohayon et al., 2016). Thus, chronic stress creates a feedback loop in which HPA overactivation and microbial imbalance reinforce one another.

Evidence from both human and animal studies supports this bidirectional connection. Individuals with anxiety, depression, and irritable bowel syndrome (IBS) often display HPA axis hyperactivity or impaired feedback regulation (Foster et al., 2021; Mayer et al., 2022; Moloney et al., 2016; Simpson et al., 2021). Similarly, germ-free mice exhibit exaggerated HPA responses to stress, which can be normalized through microbial colonization or probiotic administration, highlighting the role of gut microbes in shaping stress responsivity (Cryan et al., 2019; Sudo, 2015; Sudo et al., 2004). These findings highlight the essential role of gut microbes in shaping stress responsivity and suggest that the HPA axis is both a mediator and a target of microbiota-related interventions. Taken together, the HPA axis represents a key pathway through which stress exerts its effects on both the brain and the gut. Interventions that reduce HPA overactivation, including psychotherapy, mindfulness, and microbiome-targeted treatments, may help break the cycle of stress-induced dysbiosis and support both psychological and physical health (Mayer et al., 2022; Schnorr et al., 2014). Overall, Foster et al. (2021) concluded that the microbiota–gut–brain axis represents a dynamic and reciprocal system critical to mood regulation. Recognizing this bidirectionality highlights the need for integrative treatment strategies that address psychological, biological, and behavioral factors in the management of mood disorders (Cryan et al., 2019; Morais et al., 2021).

#### 2.2.2. Stress

Psychological factors such as stress, mental health conditions, and past maternal trauma can influence the gut microbiota and function (Cryan et al., 2019; Liu et al., 2025; Morais et al., 2021). Acute and chronic stressors can result in alterations to gut microbiota composition and function, which may subsequently impact neurotransmitter production and gut permeability (Cryan et al., 2019). Altered microbial profiles can lead to changes in the production of key neurotransmitters such as serotonin and dopamine, both central to mood regulation and stress response (Allen et al., 2017; Morais et al., 2021; Smith et al., 2023). Specifically, stress and the HPA axis play a crucial part in gut functioning. When stress is perceived, the hypothalamus releases corticotropin-releasing hormone (CRH), which stimulates the anterior pituitary gland to secrete adrenocorticotropic hormone (ACTH), which in turn prompts cortisol release from the adrenal cortex (Allen et al., 2017; Mayer et al., 2022). This cascade mobilizes energy, modulates immune responses, and prepares the body to cope with stress. While acute HPA activation is adaptive, chronic stress leads to dysregulated cortisol signaling, which can have widespread negative effects on health.

Cortisol, the primary stress hormone, plays a central role in modulating gut barrier integrity and is intricately linked to both gut microbiota and mental health outcomes (Allen et al., 2017; Cryan et al., 2019). Under normal conditions, the intestinal barrier selectively permits nutrient absorption while preventing the entry of pathogens, toxins, and antigens into systemic circulation. Chronic elevations in cortisol, however, can disrupt this protective function by impairing the structure and function of tight junction proteins, including claudins, occludins, and zonula occludens-1, which maintain epithelial cell cohesion (Moloney et al., 2016). Reduced tight junction integrity increases intestinal permeability (i.e., leaky gut) allowing bacterial components such as lipopolysaccharides to enter the bloodstream and trigger systemic inflammation (Maes et al., 2019).

In addition to direct effects on epithelial cells, cortisol indirectly impacts gut barrier function by altering the gut microbiota. Stress-induced increases in cortisol are associated with reductions in beneficial bacterial populations, including Lactobacillus and Bifidobacterium, which normally support barrier maintenance and anti-inflammatory signaling (Cryan et al., 2019; Mayer et al., 2022). Dysbiosis resulting from these microbial shifts further weakens the intestinal barrier and contributes to immune activation (Moloney et al., 2016). The combined effects of cortisol-induced epithelial disruption and microbial imbalance create a cycle in which increased intestinal permeability leads to systemic inflammation, which in turn can exacerbate HPA axis activation and stress responses. This feedback loop links chronic psychological stress with gut barrier dysfunction, dysbiosis, and downstream effects on both mental and physical health, including anxiety, depression, and gastrointestinal disorders (Bonaz et al., 2018; Foster et al., 2021; Morais et al., 2021; Simpson et al., 2021).

An increasing body of research has identified strong associations between gut dysbiosis and psychological concerns, mood regulation, trauma, and adverse childhood experiences (ACEs). Early-life trauma and ACEs have been increasingly recognized as critical determinants of long-term gut microbiota composition and stress responsivity (Callaghan et al., 2020; Hemmings et al., 2021). Hemmings et al. (2021) demonstrated that traumatic exposures during sensitive developmental periods can lead to persistent alterations in microbial communities, including reduced diversity and diminished populations of beneficial bacteria such as Lactobacillus and Bifidobacterium. These microbes are essential for maintaining intestinal barrier integrity, producing neuroactive metabolites like short-chain fatty acids, and supporting immune regulation. These biological mechanisms behind this correlation may involve alterations in gut permeability and subsequent inflammatory responses, which can perpetuate a cycles of dysbiosis and stress reactivity (Cryan et al., 2019; Liu et al., 2025; Louwies et al., 2019; Mayer et al., 2022). Additionally, such long-term microbial alterations may predispose individuals to metabolic and inflammatory conditions, as well as psychiatric disorders (e.g., disorders related to mood, anxiety, trauma/stress, substance use, and potentially self-harm and suicidal behaviors) in adulthood (Callaghan et al., 2020; Simpson et al., 2021). The consequences of trauma-induced dysbiosis extend to heightened vulnerability to stress-related disorders. O’Mahony et al. (2019) highlighted that early-life stress can dysregulate the microbiota–gut–brain axis, amplifying HPA axis reactivity, increasing cortisol secretion, and promoting inflammatory signaling. These biological changes are associated with elevated risk for anxiety, depression, and functional gastrointestinal disorders such as irritable bowel syndrome (IBS) (Cryan et al., 2019; Mayer et al., 2022). Both animal and human studies support these findings, indicating that trauma-induced microbial shifts may serve as mechanistic mediators linking ACEs to long-term mental and physical health outcomes (Callaghan et al., 2020; Morais et al., 2021).

Dysbiosis can disrupt the microbiota–gut–brain axis, leading to physiological changes that contribute to psychological distress (Cryan et al., 2019; Kelly et al., 2016). Altered microbial diversity has been observed in individuals with depression, suggesting that gut microbes play a role in mood regulation through multiple biological pathways, including immune activation, neurotransmitter production, and stress-response systems such as the HPA axis (Foster et al., 2021; Hemmings et al., 2021; Morais et al., 2021, Simpson et al., 2021; Valles-Colomer et al., 2019). Preclinical studies support these findings, showing that germ-free mice exhibit exaggerated stress reactivity and anxiety-like behaviors, which can be partially reversed by the introduction of specific microbial strains (Cryan et al., 2019; Kelly et al., 2016; Sudo et al., 2004). Moreover, fecal microbiota transplantation from individuals diagnosed with major depression into rodents has been shown to induce depressive-like behaviors, providing causal evidence for the role of dysbiosis in mood regulation (Kelly et al., 2016). These results underscore the importance of gut microbiota in shaping emotional and behavioral health.

Kelly et al. (2016) also emphasized the therapeutic potential of microbiome-targeted interventions, such as probiotics, prebiotics, dietary modifications, and psychobiotics—microbes with specific benefits for mental health. Additionally, preclinical studies have demonstrated that manipulation of gut microbiota can ameliorate depressive-like behaviors (Cryan et al., 2019; Dinan & Cryan, 2017; Simpson et al., 2021). While further clinical trials are needed to establish their efficacy, these approaches suggest novel opportunities to complement traditional treatments for anxiety and depression. Collectively, the evidence indicates that gut dysbiosis is not merely a correlate but may be an active contributor to the onset and persistence of mood disorders, highlighting the gut microbiome as a critical target for future mental health research and interventions (Cryan et al., 2019; Simpson et al., 2021).

Overall, evidence suggests that the gut microbiome acts as a critical interface through which early-life trauma exerts lasting effects on physiological and psychological well-being. Interventions that target microbial health—such as probiotics, diet modification, or stress-reduction therapies—may help mitigate the enduring impact of trauma and reduce susceptibility to stress-related disorders (Hemmings et al., 2021; O’Mahony et al., 2019). Recognizing the intersection of psychosocial adversity and gut microbiota underscores the importance of integrating both biological and psychological factors in preventive and therapeutic strategies for trauma-exposed populations.

### 2.3. Social Factors

#### 2.3.1. Sociocultural Influences

Social and cultural factors, including gender norms and perceptions of women’s health, play an important role in shaping dietary behaviors and gut microbiota composition. Gender norms influence food choices, eating patterns, and meal timing, which in turn affect microbial diversity and the relative abundance of key bacterial taxa (Amato et al., 2021; Koren et al., 2012; Valdes et al., 2018). For example, women may adhere to socially promoted “healthy” or restrictive dietary patterns, whereas men may consume higher quantities of protein or energy-dense foods, reflecting gendered expectations around body image and nutrition (Wardle et al., 2004; Westenhoefer, 2005). These gender-influenced dietary behaviors can modulate gut barrier function, immune regulation, and metabolic processes, contributing to sex-specific differences in microbiota composition and susceptibility to inflammation-related and metabolic disorders (Goodrich et al., 2014; Morais et al., 2021).

Cultural perceptions of women’s health and body image further influence dietary practices. Sociocultural ideals emphasizing thinness and body control have been associated with restrictive eating, dieting behaviors, and disordered eating patterns, which may reduce microbial diversity and alter the production of microbial metabolites (Bairamian et al., 2022; Simpson et al., 2021; Westenhoefer, 2005). Such changes in the gut microbiota may have downstream effects on immune function, metabolic regulation, and mood, illustrating the broader health implications of culturally driven dietary behaviors (Mayer et al., 2022; Morais et al., 2021). Additionally, cultural practices that dictate food choices, preparation methods, and communal eating behaviors can lead to divergent microbiota profiles among populations (Amato et al., 2021; Xie et al., 2024). For example, traditional Mediterranean diets, rich in plant-based foods, have been associated with a greater variety of gut bacteria compared to Western diets laden with processed foods (Riedl et al., 2017; Valdes et al., 2018; Vangay et al., 2018).

Together, these findings underscore the importance of considering sociocultural determinants when examining gut microbiota and women’s health. Gender norms and cultural pressures not only shape dietary patterns but also influence gut microbial composition, highlighting the interconnectedness of social, behavioral, and biological factors in microbiome research (Amato et al., 2021; Mayer et al., 2022).

#### 2.3.2. Socioeconomic Status

Socioeconomic status (SES) is a social determinant shaping gut microbial composition through both nutritional and psychosocial pathways. Access to nutritious foods and healthcare varies by SES, influencing long-term gut microbiota diversity and function (Heiman & Greenway, 2016; Valdes et al., 2018). Individuals with higher SES typically consume diets rich in fiber, fruits, vegetables, and fermented foods, which support microbial diversity and the proliferation of beneficial bacterial taxa, whereas lower-SES populations often rely on processed foods and refined sugars associated with reduced microbial diversity and increased pro-inflammatory species (Heiman & Greenway, 2016; Singh et al., 2017; Vangay et al., 2018). Disparities in healthcare access may further impact microbial development through differences in early-life microbial exposures, antibiotic use, and preventive care (Dominguez-Bello et al., 2019). Individuals with higher SES typically receive better healthcare access, including preventative services that can help maintain a healthy microbiome (Mayer et al., 2022; Miller et al., 2017; Xie et al., 2024). Conversely, those with lower SES might experience chronic stressors and environmental pollutants, further impacting their microbiota negatively (Mayer et al., 2022; Miller et al., 2017; Xie et al., 2024).

Economic insecurity also contributes to gut dysbiosis through stress-related mechanisms. Chronic stress associated with low SES activates the HPA axis, increases cortisol production, and disrupts gut barrier integrity, promoting inflammation and microbial imbalance (Cryan et al., 2019; Morais et al., 2021; Vangay et al., 2018). The combination of dietary limitations and stress-induced microbial alterations creates a cumulative burden on gut health, increasing susceptibility to metabolic, gastrointestinal, and mental health disorders (Mayer et al., 2022).

These findings highlight the importance of considering SES as a key factor in microbiome research. By influencing both diet and stress physiology, socioeconomic conditions shape the gut microbiota and may contribute to health disparities (Heiman & Greenway, 2016; Miller et al., 2017). Interventions that improve food security, healthcare access, and stress reduction in lower-SES populations may therefore play a critical role in promoting microbiome-mediated health outcomes.

#### 2.3.3. Interpersonal Relationships

Interpersonal relationships play a role in shaping gut microbiota through psychosocial and physiological pathways. Social support, for example, has been shown to buffer the negative effects of stress on the gut microbiome (Mayer et al., 2022; Miller et al., 2017; Slavich, 2020). Strong social connections can attenuate HPA axis activation, reduce cortisol secretion, and maintain gut barrier integrity, thereby protecting against stress-induced dysbiosis and its downstream effects on inflammation, metabolism, and mood regulation (Cryan et al., 2019; Morais et al., 2021; Slavich, 2020). By mitigating the physiological impact of stress, social support functions as a key psychosocial modulator of gut health.

In contrast, adverse interpersonal experiences such as intimate partner violence (IPV) can have deleterious effects on gut microbiota. Breit et al. (2018) reported that individuals exposed to IPV often exhibit dysbiosis, increased intestinal permeability, and heightened systemic inflammation. These changes are driven by chronic stress, HPA axis dysregulation, and associated behavioral disruptions, including altered diet and sleep patterns (Dinan & Cryan, 2017; Mayer et al., 2022). Dysbiosis linked to IPV may increase susceptibility to mood disorders, gastrointestinal dysfunction, and metabolic disturbances, illustrating how negative social environments can profoundly impact gut (Cryan et al., 2019; Morais et al., 2021).

Overall, interpersonal relationships can exert both protective and harmful effects on the gut microbiome. Positive social support mitigates stress-related microbial imbalance, whereas exposure to chronic relational stressors, such as IPV, promotes dysbiosis and inflammation. These findings highlight the importance of considering psychosocial context and relational environments in research and interventions aimed at promoting gut microbiome health (Morais et al., 2021; Mayer et al., 2022).

### 2.4. Psychotherapy and Counseling

Psychotherapy and counseling represent clinically meaningful, yet historically underemphasized, modulators of the microbiota–gut–brain axis. Within a biopsychosocial framework, counseling interventions function not only as psychological supports but also as potential biological regulators through their effects on stress physiology, emotional regulation, and inflammatory processes (Allen et al., 2017; Foster et al., 2021). Chronic activation of the hypothalamic–pituitary–adrenal (HPA) axis contributes to dysbiosis, impaired gut barrier integrity, and systemic inflammation (Cryan et al., 2019; Dinan & Cryan, 2017). Interventions that reduce perceived stress and enhance adaptive coping may therefore exert downstream effects on gut microbial composition by attenuating cortisol dysregulation, improving vagal tone, and decreasing neuroinflammatory signaling (Breit et al., 2018; Schnorr et al., 2014).

Emerging interdisciplinary perspectives suggest that psychotherapy—particularly approaches targeting trauma processing, cognitive restructuring, mindfulness, and emotion regulation—may interrupt stress-driven feedback loops within the brain–gut axis (Allen et al., 2017; Slavich, 2020). By modulating HPA axis reactivity and promoting parasympathetic activation, counseling may help restore intestinal barrier integrity and reduce stress-induced shifts in microbial diversity (Cryan et al., 2019; Foster et al., 2021). This is especially relevant for women, who, according to Blount et al. (2021) experience disproportionate exposure to caregiving burden, reproductive stress, and trauma-related adversity across the lifespan. In such contexts, unresolved psychological distress may perpetuate microbial imbalance, whereas therapeutic intervention may support movement toward eubiosis (Blount et al., 2021).

In reproductive healthcare settings, integrating counseling alongside obstetric, gynecologic, or fertility-related treatment may be particularly impactful. Periods such as pregnancy, postpartum adjustment, infertility treatment, and menopause involve simultaneous endocrine fluctuation and psychosocial vulnerability (Blount et al., 2021; Bjornsen-Ramig et al., 2024). Addressing emotional distress during these transitions may reduce stress-mediated microbial disruption and improve both psychological and physiological outcomes. Prior scholarship has emphasized the importance of integrating biopsychosocial models within perinatal and fertility contexts vulnerability (Blount et al., 2021; Bjornsen-Ramig et al., 2024) and microbiome research further strengthens the rationale for such interdisciplinary collaboration (Cryan et al., 2019; Mayer et al., 2022).

Although microbiome-targeted interventions have traditionally focused on diet, probiotics, and pharmacological strategies (Schnorr et al., 2014; Borre et al., 2016) counseling expands the therapeutic landscape by targeting upstream psychosocial drivers of dysbiosis. Framing psychotherapy as a relevant intervention challenges mind–body dualism and positions mental health care as central—not adjunctive—to comprehensive women’s healthcare (Blount et al., 2021; Foster et al., 2021; Schnorr et al., 2014). Future longitudinal and intervention-based research is needed to clarify the direct effects of specific counseling modalities on microbial composition and function; however, current evidence supports the conceptualization of psychotherapy as a modifiable pathway through which stress physiology, immune regulation, and gut microbial health may be optimized in women across the lifespan (Blount et al., 2021).

## 3. Methods

### 3.1. Review Design

This article adopts a narrative review design to synthesize and critically examine interdisciplinary literature addressing biopsychosocial influences on the gut microbiome in women’s health (Green et al., 2006). A narrative approach was selected due to the conceptual, theoretical, and methodological diversity of research in this area, which spans microbiology, neuroscience, psychology, psychiatry, endocrinology, public health, and women’s health scholarship. Given the rapidly evolving and cross-disciplinary nature of microbiome science, narrative reviews are particularly well suited to integrating biological mechanisms with psychological and social determinants of health, identifying patterns across the disparate literature, and advancing conceptual clarity in emerging research domains.

The purpose of this review was not to provide an exhaustive or quantitatively weighted synthesis of evidence, but rather to develop a theoretically informed understanding of how biological processes, psychological experiences, and social environments interact through the microbiota–gut–brain axis to shape women’s health outcomes. Emphasis was placed on identifying mechanisms linking hormonal regulation, stress physiology, trauma exposure, socioeconomic conditions, and mental health to gut microbial composition and function across women’s reproductive and post-reproductive life stages.

### 3.2. Search Strategy

A structured yet flexible search strategy was employed to identify relevant literature. Electronic database searches were conducted using PubMed, Scopus, Web of Science, and Google Scholar. Searches were supplemented by manual screening of reference lists from key empirical studies and review articles to identify additional influential sources not captured through database searches.

Search terms were developed iteratively and combined using Boolean operators. Core terms included combinations of gut microbiome, gut microbiota, women’s health, sex differences, reproductive health, mental health, depression, anxiety, stress, HPA axis, cortisol, trauma, adverse childhood experiences, biopsychosocial model, gut–brain axis, microbiota–gut–brain axis, hormones, pregnancy, menopause, socioeconomic status, social determinants, psychotherapy, counseling, and eubiosis. Broad terminology was intentionally used to capture studies in which microbiome processes were discussed implicitly within broader discussions of stress, reproductive health, immune function, or social determinants rather than as the primary focus. The search primarily included peer-reviewed articles published between 2000 and 2026, with earlier foundational studies incorporated when conceptually significant to understanding the development of microbiota–gut–brain research.

### 3.3. Inclusion and Exclusion Criteria

Studies were included if they:

1. Examined the gut microbiome or microbiota in human populations or translational animal models relevant to human health;
2. Addressed biological, psychological, or social determinants influencing microbial composition or function;
3. Included relevance to women’s health, sex-specific biology, or reproductive life stages (e.g., menstruation, pregnancy, postpartum, menopause);
4. Discussed mechanisms involving the microbiota–gut–brain axis, immune regulation, stress physiology, trauma exposure, hormonal influences, or socioeconomic conditions;
5. Were published in peer-reviewed journals;
6. Were available in English.

Studies focusing exclusively on non-human systems without translational implications, purely technical sequencing methodologies without biopsychosocial relevance, or non-peer-reviewed commentaries were excluded unless they provided foundational theoretical insight. No restrictions were placed on methodological design, allowing inclusion of qualitative, quantitative, mixed-methods, experimental, longitudinal, and conceptual scholarship.

### 3.4. Study Selection and Analytical Approach

Following title and abstract screening, full-text articles were reviewed for conceptual relevance to the aims of the review (Green et al., 2006). Given the narrative nature of this synthesis, inclusion decisions were guided by theoretical contribution, interdisciplinary relevance, and explanatory value rather than by methodological hierarchy or formal quality scoring (Green et al., 2006). Study screening, prioritization, and thematic coding were conducted collaboratively by two reviewers to enhance analytic rigor and reduce potential bias. Both reviewers independently evaluated articles for conceptual relevance to the microbiota–gut–brain axis and women’s health focus, and discrepancies were resolved through discussion and consensus. This dual-review process supported consistency in inclusion decisions and refinement of thematic categories across the interdisciplinary literature.

An iterative thematic synthesis was conducted across included studies. During repeated readings, patterns were identified across three primary domains consistent with the biopsychosocial framework:

- Biological mechanisms (e.g., nutrition, physical health, reproductive stages, immune modulation);
- Psychological processes (e.g., mental health, emotions, stress responsivity, trauma exposure);
- Social determinants (e.g., sociocultural norms, socioeconomic status, interpersonal relationships, health inequities).

Data synthesis followed principles of reflexive thematic analysis (Braun & Clarke, 2006, 2019). which is well suited for integrating heterogeneous interdisciplinary evidence. After repeated reading of full-text articles, preliminary codes were generated to capture key biological, psychological, and social mechanisms described across studies. These codes were iteratively organized into broader themes aligned with the biopsychosocial model, while allowing new subthemes to emerge inductively from the literature. Themes were reviewed and refined for conceptual coherence and relevance to the microbiota–gut–brain axis in women’s health. This approach enabled a theoretically grounded yet flexible synthesis of recurring patterns across diverse research domains.

Themes were derived using a theory-informed thematic synthesis approach guided by the biopsychosocial model (Engel, 1977). Initial deductive domains (biological, psychological, and social determinants) were established a priori. During iterative readings of full-text articles, subthemes were inductively refined based on recurring mechanisms and conceptual linkages reported across studies. This hybrid deductive–inductive process ensured that thematic categories were both theoretically grounded and empirically supported. These themes informed the organization of subsequent sections and the development of integrative conceptual categories linking dysbiosis, stress physiology, hormonal transitions, and social context. Attention was given to bidirectional pathways within the microbiota–gut–brain axis and to how psychosocial experiences may become biologically embedded through inflammatory and neuroendocrine mechanisms.

### 3.5. Theoretical Framework

This review is informed by the biopsychosocial model, which conceptualizes health as the product of dynamic interactions among biological, psychological, and social systems (Braun & Clarke, 2019). Within this framework, the gut microbiome is understood not merely as a biological entity but as an integrative mediator linking endocrine regulation, immune function, stress physiology, mental health, and environmental context. Biological perspectives emphasize nutrition, physical concerns, and immune system issues across reproductive life stages (Morais et al., 2021; Valles-Colomer et al., 2019). Psychological perspectives highlight mental and emotional wellbeing, stress exposure, and history/past trauma (Callaghan et al., 2020; Miller et al., 2017). Social perspectives examine sociocultural expectations, socioeconomic disparities, and interpersonal relationships (Cryan et al., 2019; Foster et al., 2021). Finally, the biopsychosocial tenets and the potential role of counseling and psychotherapy in modulating HPA axis activity was investigated. Together, these perspectives enable a relational and integrative analysis of how dysbiosis both reflects and contributes to women’s mental and physical health outcomes. The biopsychosocial framework guided both the selection and interpretation of literature, allowing for synthesis across traditionally siloed biomedical and psychosocial domains.

### 3.6. Reflexivity and Limitations

As with all narrative reviews, this synthesis reflects interpretive decisions regarding literature selection, theoretical emphasis, and thematic organization. Although efforts were made to draw from diverse disciplinary perspectives, the review is limited by its focus on English-language publications and by variability in microbiome methodologies across studies. Additionally, microbiome research remains rapidly evolving, and causal relationships between psychosocial factors and microbial composition are still being clarified. The narrative design does not include formal risk-of-bias assessment or quantitative weighting of evidence, which may limit reproducibility relative to systematic reviews. Nevertheless, the narrative approach and biopsychosocial framework provide a valuable foundation for advancing conceptual clarity, integrating interdisciplinary findings, and identifying priorities for future longitudinal and intervention-based research focused on women’s health and eubiosis. For representation of themes please see Table 2.

## 4. Discussion

### Moving Towards Eubiosis

Empirical evidence suggests that women’s gut microbiota influences biological, psychological, and social health indicators. Accordingly, targeted holistic care across the biopsychosocial tenets may help mitigate female risk factors and improve health outcomes across women’s reproductive and post-reproductive life stages (Cryan et al., 2019; Mayer et al., 2022; Morais et al., 2021). Prior work by Blount et al. (2021) and Bjornsen-Ramig et al. (2024) has outlined biopsychosocial risk factors associated with women’s health across the lifespan, including specialized contexts such as infertility treatment and in vitro fertilization (IVF). Integrating gut microbiome science into these frameworks further clarifies how psychosocial stressors, hormonal changes, and immune adaptations interact to shape women’s mental and physical health trajectories (Callaghan et al., 2020; Simpson et al., 2021).

Women experience distinct periods of biological vulnerability that coincide with heightened psychological and social demands. For example, during pregnancy and the postpartum period, dramatic endocrine shifts interact with immune modulation and stress responsivity, increasing susceptibility to mood disorders such as perinatal depression and anxiety (Mayer et al., 2022; Morais et al., 2021). Emerging evidence suggests that stress-induced alterations in gut microbiota during these periods may contribute to systemic inflammation, dysregulated neurotransmitter signaling, and impaired gut barrier function, potentially exacerbating both gastrointestinal symptoms and mental health outcomes (Cryan et al., 2019; Simpson et al., 2021). Similarly, women undergoing infertility treatment or IVF often experience sustained psychological stress, which may further destabilize gut microbial composition and HPA axis regulation, reinforcing cycles of emotional distress and physiological dysregulation (Hemmings et al., 2021; Mayer et al., 2022).

Moving beyond a solely biological treatment paradigm—still predominant in many medical settings—researchers and clinicians are increasingly encouraged to integrate medical and wellness models of care (Blount et al., 2021). Practical applications of this approach include combining counseling or psychotherapy with obstetric, gynecologic, or fertility-related medical interventions; emphasizing stress regulation and emotional well-being alongside physical outcomes; and adopting biopsychosocial frameworks in women’s reproductive healthcare (Cryan et al., 2019; Morais et al., 2021). Such integration is particularly relevant given evidence that psychological distress may undermine the effectiveness of biological interventions when stress-related dysbiosis remains unaddressed (Simpson et al., 2021; Mayer et al., 2022).

The expanding literature on the brain–gut–immune axis underscores the relevance of counseling and psychotherapy as biologically meaningful interventions within women’s healthcare settings. Historically, gut health interventions have focused on dietary modification, pharmacological treatment, and probiotic supplementation (Cryan et al., 2019; Dinan & Cryan, 2017; Kelly et al., 2016; Morais et al., 2021). While these strategies remain essential, they may be insufficient when chronic stress, trauma exposure, or emotional dysregulation continue to drive HPA axis activation and microbial imbalance (Callaghan et al., 2020; Hemmings et al., 2021). For instance, menopausal women often experience both psychological symptoms (e.g., mood instability, sleep disturbance) and gastrointestinal changes linked to estrogen decline, suggesting that addressing emotional and physiological stress concurrently may be necessary to restore regulatory balance (Mayer et al., 2014, 2022).

Counseling interventions that target emotional regulation, stress reduction, trauma processing, and resilience-building may exert downstream biological effects by modulating HPA activity, improving vagal tone, and reducing neuroinflammatory signaling (Bjornsen-Ramig et al., 2024; Cryan et al., 2019; Morais et al., 2021; Mori et al., 2018). In females, such interventions may support gut eubiosis while simultaneously reducing vulnerability to depression, anxiety, and stress-related somatic symptoms (Simpson et al., 2021; Valles-Colomer et al., 2019). This perspective reframes counseling not merely as adjunctive support, but as an intervention with potential relevance for gut physiology, immune regulation, and long-term disease risk.

Counseling services therefore represent a critical yet underutilized component of comprehensive women’s healthcare. By addressing emotional and psychosocial contributors to dysbiosis, counseling may help interrupt stress-driven feedback loops within the gut–brain axis and promote microbial environments conducive to eubiosis (Cryan et al., 2019; Louwies et al., 2019; Morais et al., 2021; Schnorr et al., 2014). Importantly, this biopsychosocial approach expands treatment possibilities beyond symptom management, positioning mental health care as a central mechanism for improving both psychological and physical outcomes in women (Mayer et al., 2014; Simpson et al., 2021).

Finally, the evidence reviewed highlights the importance of interdisciplinary models of care that integrate medical, nutritional, psychological, and social expertise. Collaboration among physicians, nutritionists, mental health counselors, and allied health professionals enables treatment plans that address the full spectrum of biopsychosocial determinants shaping gut health and emotional well-being (Blount et al., 2021; Bjornsen-Ramig et al., 2024; Mayer et al., 2022; Valdes et al., 2018). Such coordinated approaches are particularly critical in women’s health contexts, where fragmented or siloed care may fail to account for the interconnected biological and psychological demands women face. Integrative care models linking dietary interventions, stress management, counseling, and medical treatment may therefore offer more effective and sustainable improvements in women’s health outcomes than any single modality alone (Cryan et al., 2019; Morais et al., 2021; Valdes et al., 2018).

## 5. Limitations of Review

As with any narrative review, there are limitations. This review focused on biopsychosocial risk factors influencing gut microbiota by reviewing literature associated with biological, psychological, and social determinants of women’s health. Though many factors were discovered and discussed, the review is not exhaustive, as there may be additional factors influencing microbiome not included in this review. Furthermore, this paper includes a general review of the available literature, and the information and conclusions may not be applicable to all individuals.

## 6. Conclusions

This review advances a biopsychosocial conceptualization of the gut microbiome as a central, integrative mechanism shaping women’s physical and mental health across the lifespan. The evidence synthesized herein demonstrates that the gut microbiome is not a passive biological substrate, but rather a dynamic and responsive system that both reflects and mediates interactions among hormonal regulation, stress physiology, immune functioning, psychological well-being, and social context (Cryan et al., 2019; Mayer et al., 2022; Morais et al., 2021). Dysbiosis, therefore, should be understood not simply as a downstream consequence of illness, but as an active contributor to the development, maintenance, and recurrence of mental health disorders, metabolic dysfunction, and stress-related disease processes through pathways involving HPA axis dysregulation, neuroinflammation, immune activation, and altered vagal signaling (Cryan et al., 2019; Foster et al., 2021; Simpson et al., 2021).

Women’s health occupies a particularly critical position within this framework due to sex-specific biological processes and disproportionate exposure to psychosocial stressors. Hormonal fluctuations across reproductive life stages—including menstruation, pregnancy, the postpartum period, infertility treatment, and menopause—interact with stress responsivity and immune regulation in ways that uniquely shape gut microbial composition and resilience (Morais et al., 2021; Simpson et al., 2021). These biological transitions coincide with heightened vulnerability to mood disorders, anxiety, trauma-related conditions, and stress-sensitive somatic symptoms, underscoring the need to examine mental health and gut health as interdependent rather than parallel concerns (Callaghan et al., 2020; Kelly et al., 2016; Mehta, 2025; Simpson et al., 2021). When compounded by social determinants such as caregiving burden, socioeconomic strain, gendered health expectations, and exposure to interpersonal violence or early-life adversity, women may experience cumulative biopsychosocial risk that accelerates microbial dysregulation and perpetuates cycles of emotional and physiological distress (Hemmings et al., 2021; Miller et al., 2017).

Mental health emerges as a particularly salient dimension within this biopsychosocial model. The microbiota–gut–brain axis provides a mechanistic explanation for how psychological stress, trauma, and mood disorders may become biologically embedded, influencing gut permeability, inflammatory signaling, and neurotransmitter production (Cryan et al., 2019; Morais et al., 2021). In turn, these biological alterations may reinforce emotional dysregulation, heightened stress reactivity, and vulnerability to chronic mental health conditions (Simpson et al., 2021; Valles-Colomer et al., 2019). This reciprocal relationship challenges traditional mind–body dichotomies and calls for clinical approaches that address psychological distress not only as an outcome of illness, but as a modifiable driver of biological dysfunction. For women, whose mental health concerns are often underdiagnosed or minimized in medical settings, recognizing these bidirectional processes is essential for equitable and effective care.

From a clinical perspective, the findings reviewed in this paper strongly support the integration of counseling and psychotherapy into women’s health and gut health treatment paradigms. While dietary modification, pharmacological therapies, and probiotic supplementation remain foundational interventions, they may be insufficient when implemented in isolation—particularly in the context of chronic stress or unresolved trauma (Callaghan et al., 2020; Cryan et al., 2019; Morais et al., 2021). Counseling interventions that target emotional regulation, stress reduction, trauma processing, and resilience-building may exert meaningful biological effects by modulating HPA axis activity, improving vagal tone, reducing neuroinflammation, and supporting microbial environments conducive to eubiosis (Louwies et al., 2019; Mayer et al., 2022; Schnorr et al., 2014; Simpson et al., 2021). Framing counseling as a biologically relevant intervention positions mental health care as an essential component of comprehensive treatment strategies rather than an adjunctive or secondary service.

This expanded perspective has important implications for women’s reproductive healthcare. During periods such as pregnancy, postpartum adjustment, and fertility treatment, women frequently encounter heightened physiological demands alongside emotional vulnerability (Blount et al., 2021). Integrating mental health support within these settings may not only improve psychological outcomes but also buffer stress-induced microbial disruption, potentially reducing risk for adverse maternal and infant health outcomes (Callaghan et al., 2020; Mayer et al., 2022). Such an approach aligns with emerging evidence that maternal stress and dysbiosis may have intergenerational consequences, influencing offspring neurodevelopment and stress responsivity through microbial and epigenetic pathways (Hemmings et al., 2021; Morais et al., 2021).

More broadly, this review underscores the necessity of interdisciplinary, women-centered models of care that integrate medical, nutritional, psychological, and social expertise. Collaborative approaches involving physicians, mental health counselors, nutritionists, and allied health professionals allow for treatment plans that address the full spectrum of biopsychosocial determinants shaping gut health and emotional well-being (Blount et al., 2021; Bjornsen-Ramig et al., 2024; Valdes et al., 2018). These models move beyond siloed, symptom-focused interventions toward coordinated, prevention-oriented strategies that recognize the interconnected nature of women’s health experiences. Such integration is particularly critical for addressing health disparities, as women from marginalized or lower socioeconomic backgrounds may experience disproportionate exposure to chronic stressors that exacerbate dysbiosis and mental health risk (Heiman & Greenway, 2016; Miller et al., 2017).

Future research should prioritize longitudinal, sex-specific, and intersectional investigations examining how psychosocial interventions—including counseling, psychotherapy, trauma-informed care, mindfulness-based approaches, and stress-reduction strategies—directly influence gut microbiome composition, function, and stability (Cryan et al., 2019; Morais et al., 2021). Expanding this evidence base will be essential for refining clinical guidelines, informing training across healthcare disciplines, and supporting policy initiatives that embed mental health services within women’s healthcare systems. Ultimately, centering women’s mental health within microbiome research and clinical practice offers a powerful pathway toward advancing eubiosis, promoting emotional resilience, and improving long-term health outcomes for women across the lifespan (Mayer et al., 2022; Simpson et al., 2021).

## Figures and Tables

**Table 1:** Biopsychosocial influences on the gut microbiome.

Domain	Key Components
Biological	Nutrition; Physical Factors; Reproductive Life Stages; Immune System
Psychological	Mental and Emotional Health; Stress
Social	Sociocultural Influences; Socioeconomic Status (SES); Relationships

**Table 2:** Summary of evidence across domains.

Domain	Types of Evidence	Consistency of Findings	Methodological Strength	Overall Strength	Key Limitations
Biological Mechanisms (Physical, Nutritional, Immune System)	Germ-free animal models; fecal microbiota transplantation; immune and neuroendocrine studies; human observational research	High consistency across mechanistic pathways	Strong in preclinical models; moderate in human studies	Moderate to Strong	Limited causal inference in humans; methodological heterogeneity in sequencing; predominance of cross-sectional designs
Psychological Factors (Mental, Emotional, Stress, Trauma)	Observational human studies; psychobiotic trials; stress physiology research; animal behavioral models	Moderate consistency; replicated associations with altered microbial diversity	Moderate; small intervention samples; variable probiotic protocols	Moderate	Small sample sizes; short follow-up periods; inconsistent intervention methodologies
Social Determinants (Culture, SES, Interpersonal Relationships)	Dietary intervention; migration; stress physiology research; epidemiological studies	Moderate for diet; emerging for structural inequities	Moderate to Emerging	Emerging to Moderate	Limited longitudinal microbiome-specific studies on structural inequities; few intersectional analyses
Reproductive Life Stages (Menstrual Cycle, Pregnancy, Menopause)	Longitudinal; endocrine–microbiome interaction research; immune modulation	Moderate consistency, strongest in pregnancy-related findings	Moderate; fewer longitudinal studies beyond pregnancy	Moderate	Limited integration with psychosocial variables; underrepresentation of menopause and postpartum mental health
Psychotherapy and Counseling as Microbiome Modulators	Stress-reduction trials; HPA modulation research; theoretical integration; microbiome studies	Conceptually consistent; limited direct microbial measurement	Preliminary empirical support	Preliminary but Theoretically Strong	Scarcity of trials directly measuring microbiome outcomes following psychotherapy

## Data Availability

No new data were created or analyzed in this study.

## Abbreviations

The following abbreviations are used in this manuscript:

GI	Gastrointestinal
HPA	Hypothalamic–pituitary–adrenal
GABA	Gamma-aminobutyric acid
VNS	Vagus nerve stimulation
CRH	Corticotropin-releasing hormone
ACTH	Adrenocorticotropic hormone
IBS	Irritable Bowel Syndrome
SES	Socioeconomic Status
